# Eyes and negative phototaxis in juvenile crown-of-thorns starfish, *Acanthaster* species complex

**DOI:** 10.1242/bio.041814

**Published:** 2019-05-29

**Authors:** Camilla Korsvig-Nielsen, Mike Hall, Cherie Motti, Anders Garm

**Affiliations:** 1 Marine Biological Section, University of Copenhagen, Universitetsparken 4, 2100 Copenhagen Ø, Denmark; 2 Australian Institute of Marine Science, PMB 3, Townsville, MC QLD 4810, Australia

**Keywords:** Sensory ecology, Behaviour, Starfish, Echinoderm, Eyes

## Abstract

As a corallivore, the crown-of-thorns starfish (COTS; *Acanthaster* species complex), has significant impacts on coral mortality and community structure on tropical reefs throughout its Indo-Pacific range. COTS form aggregations which systematically move through and across reefs causing significant loss in hard coral cover. Previous work has shown that their behaviours on the reef are influenced by rheotaxis, olfaction and vision, with vision guiding adult animals to their coral habitat at short distances. As the compound eye of starfish grows throughout life the visual capacity of juvenile eyes is putatively less than for adult animals. Here we show this to be the case. Juvenile eyes have approximately the same visual field as adult eyes but significantly lower spatial resolution. They display negative phototaxis, as observed in adults, but we found no direct proof for the use of spatial resolution in this behaviour. Our results show that juveniles are able to use their eyes to locate their habitat: the coral reef. However, their putatively lower spatial resolution would make this visual task more difficult than for the adults.

This article has an associated First Person interview with the first author of the paper.

## Abstract (precis)

**Summary:** The juvenile crown-of-thorns starfish has much smaller eyes than adults do, which results in worse spatial resolution. Still, they use the eyes for negative phototaxis seeking shelter.

## INTRODUCTION

The corallivorous crown-of-thorns starfish (COTS; *Acanthaster* species complex), has decimated coral reefs across the Indo Pacific. The starfish naturally occur on these reefs in low densities although episodes of very high densities – outbreaks – have been observed since the 1960s, with increasing frequency in the past few decades (Zann et al., 1987, 1990; Birkeland and Lucas, 1990; Pratchett et al., 2014). In Australia, the Great Barrier Reef (GBR) has experienced alarming decline in coral cover, changing the community structure and diversity of hard coral at least partly as a result of COTS outbreaks (Pratchett et al., 2010; Osborne et al., 2011; Brodie et al., 2017). The impact of COTS outbreaks are additive to other drivers of reef degradation including increasing water temperature and bleaching, coastal development, and extractive marine industries such as unmitigated fishery pressure (Hughes et al., 2017). These impacts are accumulative and current evidence suggests they are contributing to the collapse of reef ecosystems, which will ultimately have severe ecological and economic consequences (De'ath et al., 2012). Based on the observed and predicted future impacts from COTS outbreaks on coral reefs there continues to be a strong focus to mitigate their populations primarily by physical elimination of individual adult COTS.

Manual control of COTS is an excessively laborious and expensive process, even with current technologies such as lethal injection (Pratchett et al., 2014). As such, there is a need to develop more innovative mitigation approaches to manage COTS populations. One such approach is through the exploitation of potential vulnerabilities of their sensory biology so as to decipher the mechanisms by which starfish detect reef habitats and migrate between them. Movement in COTS has been shown to be guided by olfaction, vision and possibly rheotaxis when navigating the coral reef structure (Ormond et al., 1973; Mueller et al., 2011; Drolet and Himmelman, 2004; Petie et al., 2016a; Sigl et al., 2016; Hall et al., 2017). Chemical cues released by acroporid corals can be detected by COTS over long distances by the olfactory system, which is putatively concentrated on the distal-most tube feet, though is also present in the normal locomotory tube feet (Barnes et al., 1970; Roberts et al., 2017). Although olfaction is an important sense-influencing starfish behaviour (Beach et al., 1975; Huxley, 1976; Ormond et al., 1976; Bos et al., 2013; Motti et al., 2018), recent research has shown that at short distances vision guides asteroids to locate suitable habitats, i.e. *Linckia laevigata* and COTS (Garm and Nilsson, 2014; Petie et al., 2016a; Sigl et al., 2016).

Most starfish have an advanced compound eye situated on the base of the distal-most tube foot at the tip of each arm (Yoshida, 1966; Penn and Alexander, 1980; Garm, 2017; Birk et al., 2018). In adult animals the eyes have between 10 and 300 ommatidia depending on species and have been shown to form true images in *L. laevigata* and COTS (Garm and Nilsson, 2014; Petie et al., 2016a). The starfish eye supports low-spatial vision; in COTS, the interommatidial angles have been measured to be approximately 8°. Interestingly, they also have the lowest temporal resolution measured in any animal eye with a flicker-fusion frequency of only 0.5 Hz (Petie et al., 2016a). Their visual field is strongly oval and measures approximately 100° horizontally and 30° vertically, which, taken together, indicate that the eyes are optimized for detecting large stationary objects, such as coral bommies rising from the sea floor (Petie et al., 2016a). The majority of behavioural and physiological data comes from adult specimens, however, there are indications that vision and behaviours like finding refuge, feeding and aggregation are different in the juvenile animals (Zann et al., 1987). Adults are well protected from predation by formidable arrays of long, sharp and toxin-laden spines, which are less developed in juveniles. Whereas adults COTS are sometimes fully exposed on the top of corals as they feed, juveniles typically remain well hidden in crevices and crannies, particularly during daylight hours (Pratchett et al., 2014). Still, during outbreaks the juveniles are also found feeding in exposed areas of the reef in the daytime (Zann et al., 1987).

In COTS, the eye continues to grow throughout the life of the animal by increasing the number of ommatidia. A juvenile COTS measuring 1.5 cm in diameter has ∼20 ommatidia, whereas larger (≥40 cm) adult specimens have eyes with ∼300 ommatidia (Petie et al., 2016a). This difference will presumably result in less acute vision in juvenile animals and impact on their ability to perform the same visually guided behaviours as the adults.

COTS have very high fecundity and large females (>35 cm diameter) release more than 30,000,000 eggs twice every year (Zann et al., 1987; Babcock et al., 2016). With high fertilization rates, spawning events from adult aggregations can potentially lead to incredibly high numbers of larvae and, if conditions are optimal, to high numbers of settled juveniles and adults. It is thus of great importance to understand their sensory ecology; specifically, how they detect each other, potential feeding grounds and cryptic refuge habitats.

In this paper, we investigate the visually-guided behaviour of juvenile COTS from the GBR through controlled tank experiments. In addition, we evaluate the quality of vision from the number of ommatidia, the visual field and the interommatidial angles all obtained through underwater goniometry. We tested the hypotheses that (1) juveniles display negative phototaxis and are attracted to dark objects even when spatial resolution vision is required to detect them and (2) the low number of ommatidia in the eyes of juveniles results in significantly greater interommatidial angles and thus lower spatial resolution than found in the adults.

## RESULTS

### Eye morphology

**Fig. 1.**
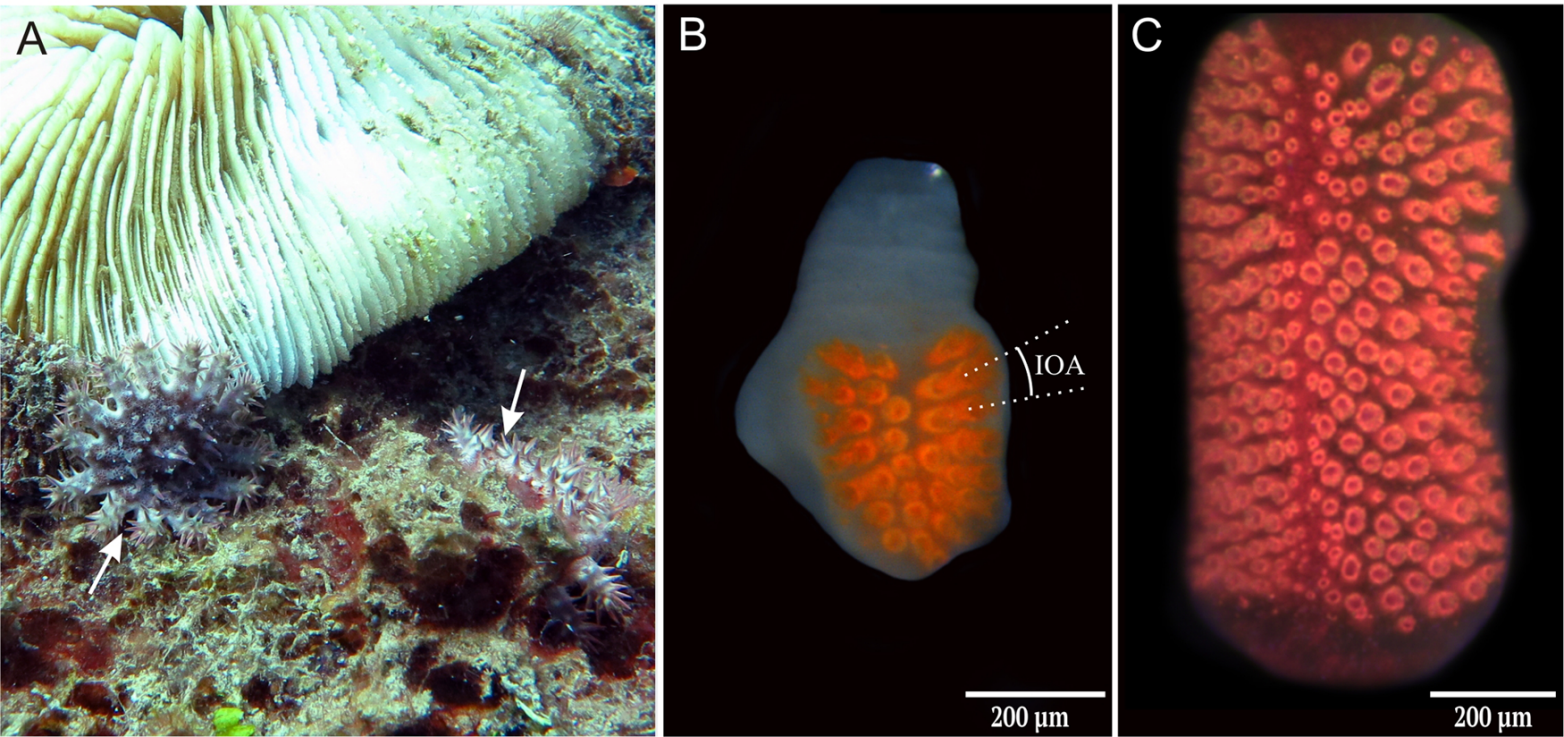
**Compound eyes of COTS.** (A) Two juvenile COTS (white arrows) sitting close to a Fungia coral. Picture was taken during daytime. Copyright AIMS/Credit: LTMP. (B) Compound eye from a juvenile with a diameter of 3.3 cm. Average eye size was approximately 260 µm in width and 350 µm in length. Spatial resolution is estimated from the inter ommatidial angle (IOA) which is the angle between the optical axes of two neighbouring ommatidia (broken lines). (C) The compound eye of an adult COTS possesses approximately 250 ommatidia. Note that the compound eye grows continuously, with the length of the eye increasing more so than the width as the number of ommatidia multiplies.

COTS have a simple compound eye located on the outer tip of each arm. The eye, sometimes referred to as the optical cushion, is positioned at the base of the distal-most tube foot. The red screening pigment from the individual ommatidia of the eye makes it clearly visible (Fig. 1). The overall structure in the juvenile eyes resembled the structure of adult eyes, though the bilateral organization and the midline were not always very clear. Four eyes from each of four animals and three eyes from a fifth animal (diameters: 3.2 cm, 3.3 cm, 3.5 cm, 3.6 cm and 5.6 cm) were size measured and had a width of 260±24 µm (mean±s.d., *n*=20) and a length of 365±36 µm. The average number of ommatidia in each eye was 26.9±2.3 and a tendency for more ommatidia with increasing size of the animal was observed (Table 1). The width of the ommatidia including screening pigment was 29±6.2 µm (mean±s.d., *n*=23).

**Table 1.**
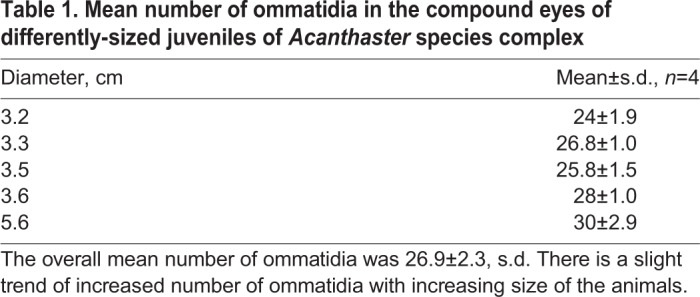
**Mean number of ommatidia in the compound eyes of differently-sized juveniles of *Acanthaster* species complex**

### Spatial resolution and visual field

Interommatidial angles were measured with an underwater goniometer and used as a proxy for the spatial resolution. Twenty-nine angles from five eyes were measured and had a mean of 16.8°±4.5° (mean±s.d.). There was no significant difference between the interommatidial angle from the different juvenile eyes (one-way ANOVA *F*_2,26_=0.597, *P*=0.558). However, when the juvenile eyes were compared with adult eyes their interommatidial angles were significantly larger (two-sided unpaired *t*-test, *P*<0.0001, *n*=29 and 50 respectively). Goniometric measurements were also used to map the visual field of the eyes; horizontally it was 118°±10° and vertically it was 37°±5° (mean±s.d., *n*=5).

### Behaviour experiments

The visual capabilities of the animals were tested in a circular behavioural arena using two different sets of visual stimuli testing for negative phototaxis and spatial vision, respectively. The results from both experiments were analysed using circular statistics (Table 2). When the animals were transferred to the behavioural arena, they initially curled up. After 0.5–1 min they would unfold in a random direction and typically start walking in that direction.

**Table 2.**
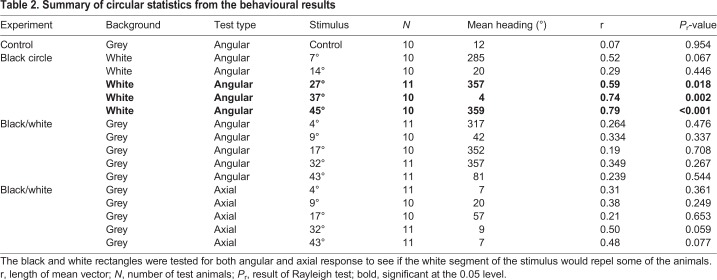
**Summary of circular statistics from the behavioural results**

**Fig. 2.**
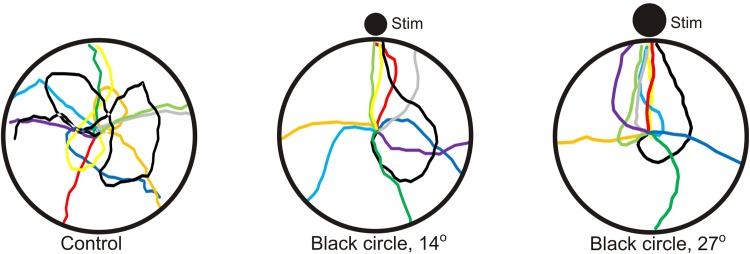
**Examples of trajectories from behavioural experiments.** (A) In the control experiment with no visual stimuli, the juvenile COTS walked randomly in the arena. (B) When presented with a black circle with an initial angular height of 14° on a white background, the animals still did not show significant attraction to the stimuli (see Table 2 for statistics). (C) Animals presented with a larger black circle (initial angular height 27°) were attracted to the stimuli (*P*=0.018, see Table 2 for details).

**Fig. 3.**
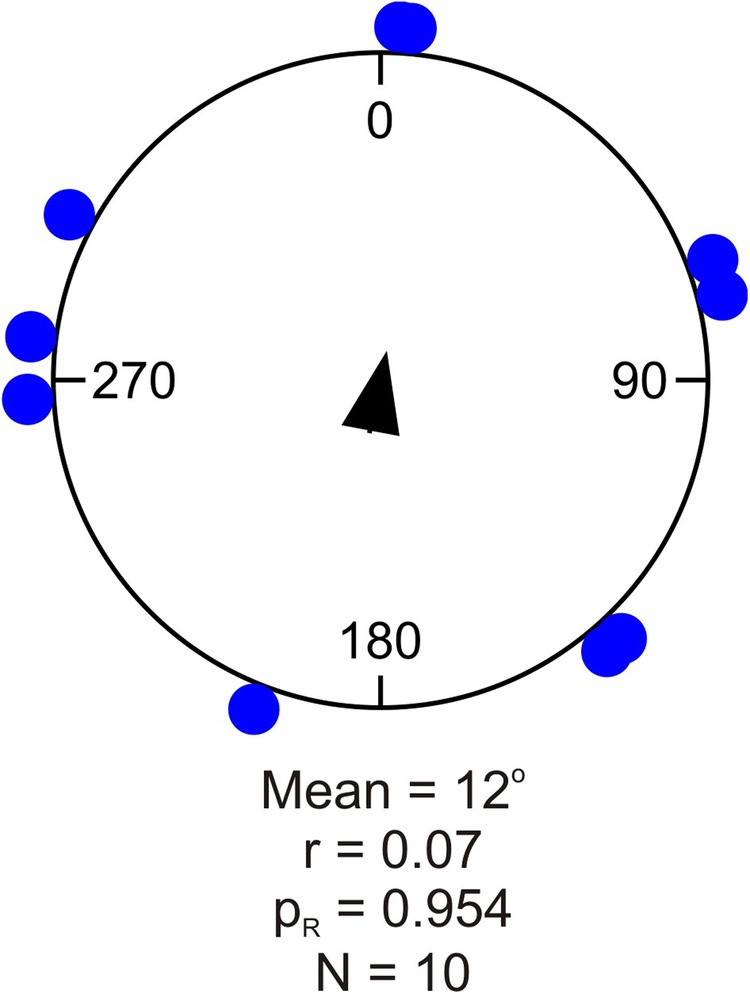
**Circular statistics from the control experiment using an evenly grey background without visual stimuli.** The blue dots represent the angular position where individual animals contacted the arena wall. The mean heading (°) of the animals is indicated by the mean vector (central arrow). In these experiments the animals displayed no directionality and walked randomly in the arena (*P*=0.954). A summary of the circular statistics is given in Table 2. r, length of mean vector; *N*, number of test animals; *P*_R_, result of Rayleigh test.

In the control experiment, without any visual cues, the animals walked randomly (Rayleigh test, *P*=0.954, Figs 2 and 3). The walking trajectories had a mean length of 152 cm±130 cm (mean±s.d., *n*=10) (Table 3). There was no preferred side or region of the tank in absence of visual cues.

**Table 3.**
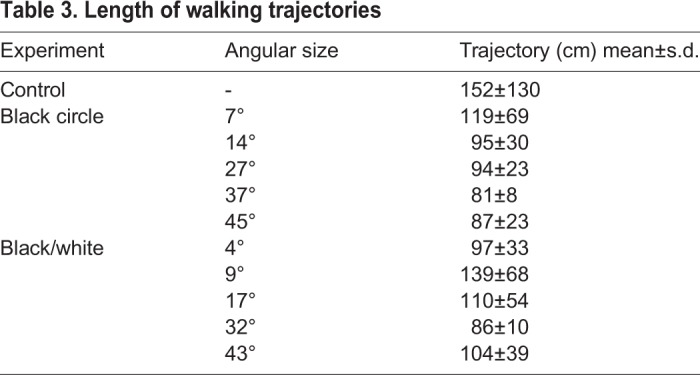
**Length of walking trajectories**

**Fig. 4.**
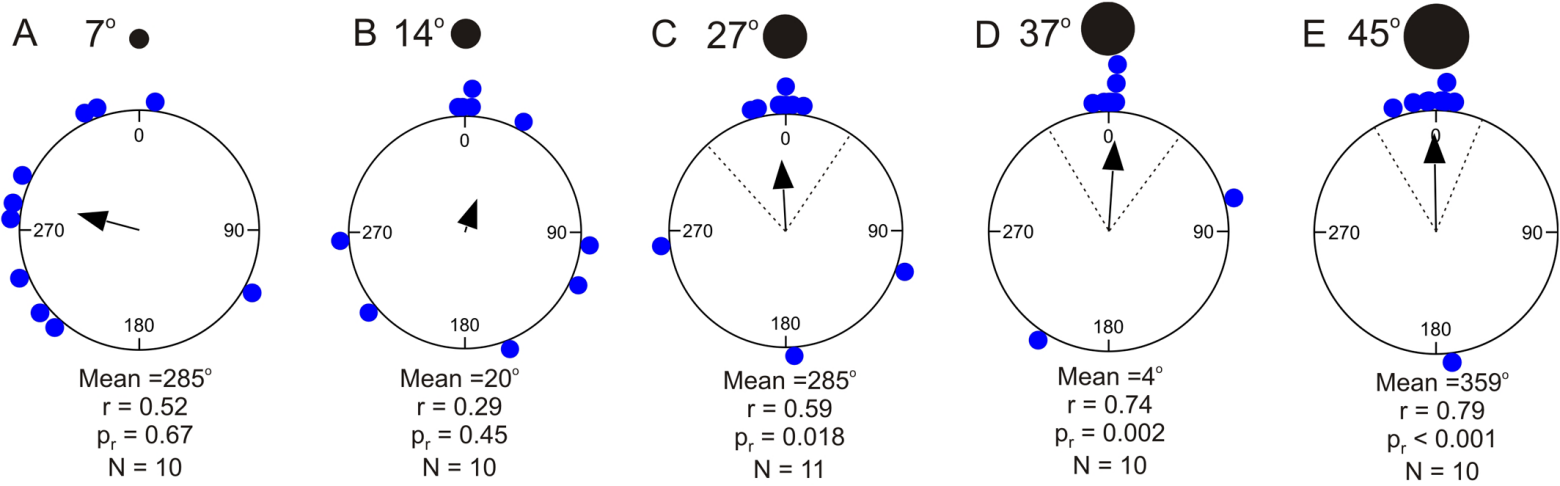
**Circular statistics of behavioural experiments using black circles on a white background.** Black circles of different sizes were placed on the bottom at a randomly chosen position along the wall of the behavioural arena. The position of the stimulus was changed between each trial (always set to position 0 in the plot). (A,B) Animals were not attracted to stimuli with an angular height of 7° and 14°. (C–E) When presented stimuli of 27° or larger, the animals were attracted and walked towards the stimulus. See also Fig. 2. A summary of the circular statistics is given in Table 2. r, length of mean vector; *N*, number of test animals; P_r_, result of Rayleigh test. Dashed lines indicate 95% confidence intervals.

To test for negative phototaxis, the juvenile COTS were presented with black circles on a white background providing high contrast stimuli. When presented with the two smallest stimuli the starfish walked randomly in the arena (Figs 2 and 4, Table 2). The starfish were attracted to the three largest stimuli with angular heights of 27° or higher (Figs 2 and 4, Table 2, Rayleigh test *P*<0.018, stimuli included in 95% confidence interval, *n*=10 or 11 for all stimuli). The trajectory lengths varied between 81 and 119 cm (Table 3) but there were no significant differences in trajectory length between any of the black circles (one-way ANOVA, *F*_10,101_=1.685, *P*=0.094; Fisher’s LSD post-hoc test, 0.12<*P*<0.97). Still, the four larger stimuli resulted in significantly shorter trajectories than in the control experiment (one-way ANOVA, *F*_10,101_=1.685, *P*=0.094; Fisher’s LSD post-hoc test, *P*<0.02).

**Fig. 5.**
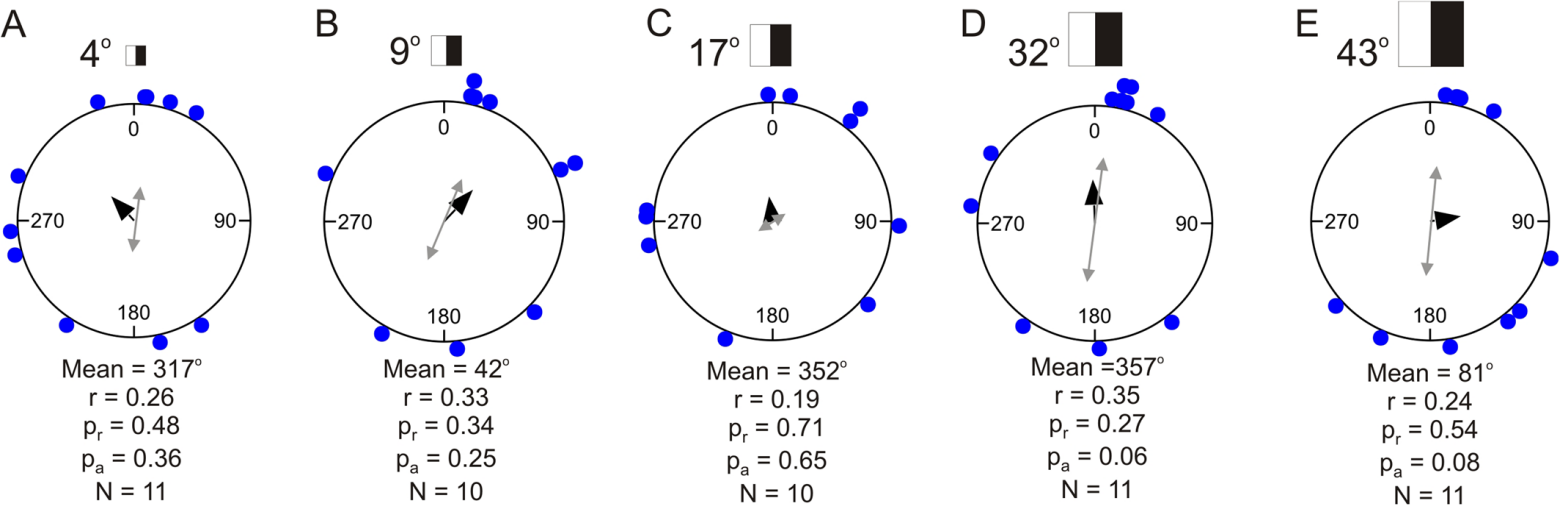
**Circular statistics of behavioural experiments using paired white and black rectangles (black always to the right) on grey background.** The average intensity of the rectangles matched the grey background and they can thus only be detected using spatial vision. Stimuli of different sizes were placed on the bottom at a randomly chosen position along the wall of the behavioural arena. The position of the stimulus was changed between each trial (always set to position 0 in the plot). (A–E) Animals were not attracted to any of the five differently sized stimuli when the testing with unidirectional circular statistics. Using an axial test, there was a strong tendency for a directional response to the two largest stimuli (grey double arrows). For a summary of the circular statistics, see Table 2. r, length of mean vector; *N*, number of test animals; P_r_, result of Rayleigh test; *P*_a_, result of axial Rayleigh test.

When presented with the white and black rectangles on a grey background the juvenile COTS showed no significant directional walking (Figs 3 and 5, Table 2, Rayleigh test, *P*>0.267, *n*=10 or 11 for all stimuli). Still, it was seen that for the larger stimuli, some of the starfish seemed to be attracted and moved directly toward the black (right) side of the stimulus (Fig. 5). Since the stimuli also contained a bright (white) side it is possible that this part repels the animals, therefore, to test for this, a modified Rayleigh axial test was conducted (Table 2). Although the axial test also returned no significant directionality in the behaviour of the starfish there were strong indications for the animals responding to the stimuli of 32° (*P*=0.059) and 43° (*P*=0.077) (Table 2). The trajectory lengths varied between 86 and 139 cm (Table 3) but there were no significant differences in trajectory length between any of the black and white rectangles (one-way ANOVA, *F*_10,101_=1.685, *P*=0.094; Fisher’s LSD post-hoc test, 0.08<*P*<0.82). Regardless, the 4° and 32° high rectangles produced significantly shorter trajectories than the control experiment (one-way ANOVA, *F*_10,101_=1.685, *P*=0.094; Fisher’s LSD post-hoc test, *P*=0.023 and 0.008, respectively).

## DISCUSSION

The function of the compound eye in the starfish *L. laevigata* and COTS has recently been linked to their ability to navigate towards suitable habitats and feeding grounds within relatively short distances (Garm and Nilsson, 2014; Petie et al., 2016a,b; Garm, 2017). These findings are of ecological relevance, especially in regards to the potential to manage or mitigate the reoccurrence of COTS outbreaks. As all previous data comes from adult specimens this study investigated the compound eye and vision in juvenile COTS with the aim to understand the importance of vision throughout their lifecycle. Our findings imply that, despite having significantly fewer ommatidia and lower spatial resolution, juveniles are capable of using vision to locate suitable habitats and potential feeding grounds as the adults do. The juveniles were able to detect dark structures on a bright background displaying negative phototaxis. The morphological results confirmed the structural basis for low spatial resolution vision, but the behavioural experiments gave no direct proof, only strong indications for this that might be a consequence of the sample size.

### Quality of vision

It is a well-documented fact that the vision in some animals changes with age and, in general, achieves higher acuity as the juvenile matures (Carvalho et al., 2002; Evans and Fernald, 1990; Meyer-Rochow, 1975; Cronin, 1986). Our results show that this is also the case for COTS*.* Firstly, we corroborate the results from Petie et al., 2016a, that the number of ommatidia increase as the animal grows. We found that the juvenile compound eyes included in the current study had about ten times fewer ommatidia than the adult eyes (24–30 ommatidia versus 250–300 ommatidia). Fewer ommatidia will either result in a smaller visual field, a lower spatial resolution, or both. We find that it is only the spatial resolution that changes since the juvenile eye has a visual field of 37° vertically and 118° horizontally – more or less identical to the adult eye (Petie et al., 2016a). On the other hand, the spatial resolution increases with age, inferred from the interommatidial angle, which changes from 17° to 8°. This finding can be reconciled with the observed life stage transition; juvenile COTS (<20 cm) remain highly cryptic during the day and favour nocturnal feeding, whereas adult COTS (>40 cm) exhibit weak diurnal feeding activity patterns and show little tendency for cryptic behaviour (Keesing, 1995). A similar change is found in the visual acuity of the stick insect, *Carausius morosus*, with an increase in ommatidia from <300 to >600 as they transition from juvenile to adult (Frolov et al., 2012). In stick insects, a concomitant increase in light sensitivity also follows with major impact on lifestyle. The light sensitivity of the juvenile stick insect is inadequate for nocturnal behaviour, forcing them to be diurnal, but sensitivity increases with each moult and enables the adult insect to lead a safer nocturnal lifestyle (Frolov et al., 2012).

In COTS, the single ommatidia seem to vary little with age and maintain a similar size and pupil diameter. This indicates that sensitivity is the same and that the developmental program for ommatidia is relatively strict and stable throughout life, similar to many insect compound eyes (Niwa et al., 2004). Even though we do not have specific data on the density of the outer segments in juvenile ommatidia our behavioural data strongly suggest that the light sensitivity is the same in juveniles and adults. Further, since the number of arms and thus eyes does not change after reaching the age of 5 months (1–1.5 cm) (Yamaguchi, 1974) and since the juvenile eye has the same visual field as the adult eye, the complete visual field of the entire animal is the same past the age of 5 months. With 20–25° between each arm and with a horizontal visual field of ∼100° there is significant overlap between neighbouring eyes. Complementing this, the adult COTS eye has the lowest temporal resolution published for any animal (Petie et al., 2016a), a trait which potentially greatly enhances light sensitivity. This suggests COTS are capable of visually guided nocturnal activity, however, there is no direct evidence for this in either adults or juveniles.

### Negative phototaxis and image formation

One of the simplest visually guided behaviours is phototaxis – orientation towards light gradients (Dusenbery, 1992; Nilsson, 2013). We tested negative phototaxis in juvenile COTS using high-contrast (∼1) black circles on a white background. Juveniles displayed negative phototaxis when the dark object had an angular size of 27° or more, whereas the adults responded at 14° (Petie et al., 2016b), which corresponds to their higher spatial resolution. Using the black and white stimulus on the grey background revealed another difference; the juveniles did not display a significant axial response as the adults did. For the juveniles, the axial test only revealed a strong tendency for directionality when using the two largest stimuli (Table 2). As such, the results indicate the usage of true image information in juveniles but with no direct proof. In support of the indication though, the trajectories obtained with the 32° rectangles were straighter (shorter and more direct, Table 3) than for the control and all the individuals that did reach the 32° and 43° stimuli made contact with the black segment. Based on the estimated spatial resolution (1–2×interommatidial angle), the largest stimuli should be readily detected by the juveniles. Their lack of attraction to the two largest stimuli could thus infer that at least in juvenile COTS image information is used for behaviours other than negative phototaxis. It is not uncommon that animals display a number of visually-guided behaviours requiring different degrees of spatial resolution (Wehner, 1987). Recent work on deep-sea starfish suggests that here vision might be involved in food detection and intraspecific communication, both in combination with bioluminescence (Birk et al., 2018).

### Visual ecology

The present study indicates that juvenile COTS of 3–5 cm in diameter have similar visual ecology as the adult animals. They have the same horizontally-elongated visual field indicative of their interest in objects rising from the sea floor. They also display negative phototaxis, which guide them to the reef structures within their habitat (Petie et al., 2016a). Newly settled juvenile COTS feed on coralline red algae (Zann et al., 1987) and, in the presence of an adequate food supply, will move very little. They transition into corallivores at ∼6 months of age and at a size of 1–2 cm (Yamaguchi, 1974; Zann et al., 1987; Johansson et al., 2016), at which point they actively seek out coral prey. This fits well with all the juveniles in this study (3–5 cm) feeding readily on hard coral tissue and it would be interesting to test the visual capacity of juveniles prior to the transition. Their putatively less-active lifestyle may require little in terms of visual capacity.

Adult COTS and *L. laevigata* are both capable of visually detecting their coral habitat and in this way navigate towards it, albeit over rather short distances of a few meters (Garm and Nilsson, 2014; Petie et al., 2016a; Sigl et al., 2016). Vision is thus not likely involved in guiding the animals to new reef habitats but rather to make sure they do not move away from the reef they are already on (Petie et al., 2016a). This is in accordance with the general notion that adult COTS do not normally move away from their settlement reef unless physically displaced e.g. by currents or a cyclone (Zann et al., 1987). The lower spatial resolution demonstrated here shows that juveniles are even less likely to move off their home reef. This corroborates a previous study where the juveniles were found to move much less than the adults, be mainly nocturnal and in general displayed a more cryptic behaviour (Zann et al., 1987). Still, the results here show that already as juveniles, COTS possess eyes which have the morphological requirements to form images and are potentially involved in habitat detection.

## MATERIALS AND METHODS

### Animals

Juvenile COTS were collected by Reef Magic Cruises from the GBR off the coast of Cairns, Australia during January 2017. A total of 13 animals (mean diameter: 4.3 cm, min: 2.9 cm, max: 5.5 cm; s.d., 0.74; 13–19 arms per animal) were transported in an aerated plastic container to the Australian Institute of Marine Science (AIMS) where they were transferred to a 750 l holding tank with flow-through seawater mimicking the collection site (temp, 24°C; salinity, 35‰; dark:light cycle of 11:13 h). Dark plastic tubes, mimicking cryptic refuges, and a 15×15 cm live coral colony (*Acropora* sp., as a food source) were added to the holding tank. Hereafter they were fed small pieces of coral every alternate day. Animals were acclimatized for 6 days before commencing behavioural studies.

### Behavioural arena

Behavioural studies were conducted in an indoor tank (diameter, 4 m) containing a submerged circular behavioural arena with a diameter of 160 cm (see Petie et al., 2016b for details). When not running experiments, the water level in tank was 5–10 cm above the edge of the arena to ensure adequate water flow and water exchange. A full spectrum plasma lamp [Luxim (LUMA) STA-40] lit the arena from above providing a close-to-even illumination (2700 lux at arena centre; 2370 lux at perimeter). The different visual stimuli (see below) were positioned by attaching them with Velcro to the base of a transparent Plexiglas plate (80×100 cm) which was in turn attached by clamps and grey tape to the wall of the behavioural arena. The floor of the arena was marked with a 20 cm grid to allow measurements of walking speed and the centre starting position was marked with a cross. Once animals were positioned on the centre mark, behavioural experiments were considered as initiated.

### Stimuli

Two types of visual stimuli in five different sizes were used. The first type, used to test phototaxis, was black circles used against the white wall of the behavioural arena optimizing contrast. The five black circles tested were 7°, 14°, 27°, 37° and 45° in angular height and width, as seen from the centre mark. Circles were chosen for the single object stimuli as their angular size is independent on the orientation of the visual sampling by the eye. The second type, used to test spatial resolution, was equally sized black and white rectangles immediately adjacent to each other forming a square, always with the black rectangle to the right. The rectangles were tested against a grey arena wall. The reflected radiance of the grey equates precisely to the averaged reflected radiance of the black and white rectangles resulting in the stimuli only being detectable by means of true image forming vision (see Petie et al., 2016b for details). The rectangles were 4°, 7°, 17°, 32° and 43° in angular height, as seen from the centre mark. Rectangles were chosen here over circles to avoid grey areas between the two parts. All visual stimuli were printed on waterproof vinyl, as were the white and grey backgrounds (Lotsa-Print & Signage, Townsville, Australia).

### Experimental protocol

All behavioural experiments were conducted in January 2017. The 10 different stimuli were tested in random order. Once a stimulus was positioned the response of 10 or 11 randomly chosen animals were tested one at a time. Each animal was size measured before being positioned at the centre mark and time-lapse recordings (see below) were initiated upon positioning. A successful trial ended when the experimental animal reached the arena wall. A trial was ignored if the animal did not reach the wall within 1 h as they were considered inactive (normal animals reached the wall within 10 min, including those not going for the stimuli). Six out of the total 120 trials were discarded in this way. The same stimulus was used until at least ten successful experiments were completed after which the stimulus was changed. Each animal was only used once for each stimulus safeguarded by using two separate holding tanks; one for the animals tested with a given stimulus and one for those not yet tested. The position of the visual stimuli along the wall of the arena was selected randomly and changed between each experiment. Further, the entire arena was rotated after each stimulus to avoid potential direction preferences.

### Data recording and analyses

Time-lapse images were recorded with a GoPro Hero 3+ camera inside a waterproof case and controlled with an iPhone 7 using the application ‘Capture’. The images had a 5 s interval and were afterwards converted into a video using GoPro Studio (v2.5.10). ImageJ (Ver. 1.51 Wayne Rasband, National Institute of Health) was used to track the total distance the animals moved from starting point to the arena wall and the overall directionality of the response. The directionality was defined as the angular position from the centre mark to where the animal contacted the wall, with the centre of the stimulus being set to 0. This was chosen over their initial heading since this was normally set by their random direction of unfolding (COTS curl up into a ball for protection when handled). The length of the trajectories were measured in ImageJ (Ver. 1.51, Wayne Rasband, National Institutes of Health).

### Light microscopy

Four arm tips were removed from five juvenile COTS using a pair of fine scissors. The arm tips were fixed in filtered seawater containing 4% paraformaldehyde for transportation to University of Copenhagen after which they were transferred to 0.1 M phosphate buffered saline (PBS). Eyes were dissected from the arm tips using a pair of fine scissors and were photographed using a Leica MZ 9.5 dissection microscope equipped with an Evolution MP 5.0 (Media Cybernetics, Inc., Rockville, MD 20850 USA). From these images the eye size was measured and the number of ommatidia on each eye counted. Only ommatidia with a clear pupil were included in the counts.

### Goniometry

Five eyes taken from a total of three specimens (diameters: 3.3 cm, 3.6 cm and 5.6 cm) were placed separately in a custom-made underwater goniometer and observed under a standard dissection microscope. Due to limitations within the goniometer, it was not possible to measure the visual field of the entire eye, thus the bilateral symmetry was used and only half the visual field was measured which was then mirrored to obtain the complete visual field. The optical axes of five to six ommatidia evenly distributed along the periphery of the one side were used to define the outline of the visual field. The optical axis was found by adjusting the goniometer to centre of the round ommatidia, which could be done with an accuracy of 1–1.5° determined by repeated measures of the same ommatidium. The interommatidial angles (angles between the optical axes of neighbouring ommatidia) were used as a proxy for the spatial resolution. An ommatidium was selected at random and the optical axis was measured along with the optical axes of the neighbouring ommatidia (typically three or four). The interommatidial angle was then found using the cosine formula for a sphere:
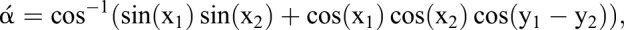
where x_1_, y_1_ are the angular coordinates of the centre ommatidium (set to 0,0) and x_2_, y_2_ are the angular coordinates of the neighbouring ommatidium and ά is the interommatidial angle. This was done for one or two ommatidia on each of the five juvenile eyes and a total of 29 interommatidial angles were measured.

### Statistics

The directionality of the trajectories was tested using circular statistics in Oriana 4 (Kovach Computing Services, UK). First, a Rayleigh test was performed to test for directionality and, if a significant directionality was found, it was further tested whether the 95% confidence interval of this direction included the centre of the stimulus. In the case of the black and white rectangles, both unidirectional and axial tests were carried out. The average trajectory lengths for each stimulus were compared using a one-way ANOVA followed by a Fisher’s LSD post-hoc test (BioStatPro 6.2.5., Analystsoft Inc., CA, USA). A two-way unpaired Student’s *t*-test was used to compare the interommatidial angles between juveniles and adults using data for adult animals, five eyes and 50 angles, from Petie et al. (2016a). The critical *P*-value was set to 0.05 for all tests.
